# Omics Biology in Diagnosis of Diseases: Towards Empowering Genomic Medicine from an Evolutionary Perspective

**DOI:** 10.3390/life14121637

**Published:** 2024-12-10

**Authors:** Emanuel Maldonado, Imran Khan

**Affiliations:** 1 CICS-UBI—Health Sciences Research Center, University of Beira Interior, 6201-506 Covilhã, Portugal; 2 MIBS Research Programme, Faculty of Biological and Environmental Sciences, University of Helsinki, 00014 Helsinki, Finland; imran.khan@helsinki.fi

## 1. Recalling the Special Issue Aims and Scope

In this section, we reintroduce the original aims and scope of the Special Issue entitled “Omics Biology in Diagnosis of Diseases: Advances in Bioinformatics and Data Analyses”, enabling readers to find an appropriate framing for the remainder of the present closing editorial. Readers aware of this can skip this section.

“The study and diagnosis of many human diseases is often difficult due to several factors. Diseases have diverse causes, such as, for instance, interactions with the environment, food habits, microbiome and/or genetics. Specifically, in this latter case, we are chiefly concerned with hereditary genetics, in particular, with the identification of the causes that induce the symptoms presented by the patients. Here, the physician often requires the collection of samples, something which will allow the performance of the genetics diagnosis. These samples often undergo the process of sequencing (i.e., whole-exome sequencing—WES) which enables the detection of deleterious genetic changes and other variation found in the protein-coding regions of the human genome. However, the genetic causes may also be located outside of the coding regions, i.e., in noncoding DNA, which comprises more than 90% of the human genome. Here, the whole-genome sequencing (WGS) methods become necessary. Additionally, GWAS, epigenomics, transcriptomics, and other omics can reveal themselves to be useful in the study and molecular diagnosis of human diseases.

Moreover, several bioinformatics advances have enabled the analysis of data originating from these sequencing technologies and often benefit from tools and methods previously developed in the context of evolutionary and comparative genomics.

This issue of Life invites authors to publish original research on all aspects of (i) genome biology and (ii) human molecular disease diagnosis (WES, WGS, etc.), with preference for articles in the former, demonstrating the (potential of) application to the latter. Potential areas for consideration include: (1) bioinformatics software and methods, possibly incorporating evolutionary and/or comparative omics aspects; (2) analyses of cohorts suspected of rare genetic diseases considering the identification of its causes; (3) omics analyses involving the human and/or other evolutionarily close species; and (4) manuscripts considering the analyses of human microbiome (pathogens) or other factors with potential to influence the human genetics and/or cause disease.” [1].

## 2. An Overview of Published Articles

The contributions to this Special Issue are listed in Section *List of Contributions,* and are hereafter briefly overviewed.

The first contribution, from Athar M et al., focuses on investigating genetic variants associated with Familial Hypercholesterolemia (FH), specifically exploring the pathogenicity of these variants and their genotype–phenotype correlations. The authors utilized whole-exome sequencing (WES) and comprehensive downstream bioinformatics analyses to achieve their objectives. Their research involved a three-generation Saudi family affected by FH, leading to the discovery of a novel frameshift variant in the LDLR gene and a single nucleotide variant in the APOB gene. These findings contribute to the growing catalog of FH-related variants, enhancing our understanding of the disease and opening new avenues in the development of novel treatments.

The second contribution, from Chou PC et al., is a comprehensive review that highlights epigenetic markers associated with Post-Traumatic Stress Disorder (PTSD) and the intergenerational inheritance of trauma. PTSD, a psychiatric disorder, is characterized by significant functional impairments and is linked to both personal experiences and familial transmission across generations. This phenomenon can be attributed to mechanisms of epigenetic inheritance, which play a pivotal role in mediating the biological effects of trauma and stress. The review provides valuable insights into the molecular underpinnings of PTSD and the complex processes through which trauma is biologically transmitted across generations.

The third contribution, from Kumar AHS, explored the protein–protein network of human SORT1 and its targetability by using pharmaceuticals. The authors identified several high-affinity interaction networks involving the SORT1 regulation of tissue fibrosis or the microcalcification process due to its influence in several biochemical processes. They also found 12 potential drug interaction sites with varying scores and probabilities in SORT1 binding pocket analysis, of which 5 were targetable in a feasible concentration of small molecules. Overall, SORT1 is involved in relevant networks and can be targeted using currently approved small-molecule therapeutics or nutraceuticals, potentially reducing the cardiac/vascular microcalcification process.

The fourth contribution, from Dsouza NR et al., combines in a single study the somatic and germline variants of PI3K enzymes known to regulate cell growth and differentiation. The somatic variants are associated with cancer and drive a proliferative phenotype, whereas the germline variants are associated with different phenotypes. Through the use of molecular dynamics simulations, the study deciphered how different variants in PIK3R1 affect opposing phenotypes, such as undergrowth and overgrowth. The variants associated with undergrowth destabilize molecular interactions with the PIK3CA receptor, and overgrowth variants lead to loss of inhibitory interactions. Regardless, all disease variants show dysfunctions on either structural characteristic and/or intermolecular interaction energy.

In the fifth contribution, from Kim S et al., the Genome-Wide Association Studies (GWAS) approach is applied to analyze genetic variants associated with sensitive skin (SS) in a set of 1690 Korean female patients. The authors uncovered genetic variations related to oxidative stress, cell growth regulation, and neurobiology. These phenotypes potentially influence skin sensitivity and provide a basis for further research.

## 3. Discussion

Personalized medicine is a recent medical model that seeks to improve patient health by characterizing and correlating an individual’s genotype and phenotype [2]. This allows us to identify appropriate and time-sensitive therapeutic strategies for each patient, to determine their predisposition to disease, and/or to deliver timely and targeted prevention [2]. This is likely to be of use for patients suffering from tumorous or cancerous diseases or from other rare (hereditary) diseases. Such diseases are frequently framed as being (i) complex due to the involvement of several genes and affected pathways, or (ii) the simpler case of monogenic diseases [3,4,5,6]. Given the ever-increasing focus on the study of tumorous/cancerous diseases, often somatic in nature, we have decided to focus this Special Issue on cases of hereditary rare diseases, which often receive less attention.

### 3.1. Recent Developments

Several omics approaches provide particular views of biological systems, enabling the study of diseases from different perspectives and allowing us to identify their causes at different biological levels, thereby providing a better understanding of health and disease. Detailed characterization of an individual’s genotype can be achieved by employing sequencing technologies, which often involve several methods, such as whole-genome sequencing (WGS) and whole-exome sequencing (WES) [3,7]. The former allows us to acquire a complete view of the genome, with access to the both noncoding and protein-coding complete sub-genomes, while the latter by being based on a targeted sequencing method is aimed at protein-coding regions [7,8]. Both methods allow for the detection of (deleterious) genetic changes and variations correlated to health and disease states. The technology often involved in these methods is essentially related to first- and second-generation technologies (often termed next-generation sequencing—NGS) [7,9]. A third generation attempted to improve the results of its predecessor, but still presents less accuracy [7,9]. However, such progress has been important to improve the completeness of whole-genomes by combining short and long sequencing technologies, thus improving the potential for the detection of additional variation [4] and the accuracy of complete assemblies [10]. Following the emergence of NGS with the establishment of high-throughput and less time-consuming activities [9], the first-generation technology became useful for corroborating bioinformatics results and hence ensure that they are accurate and can be safely reported back to the patients (e.g., [11]).

The epigenetics approach [12] has implications in detecting how genes interact with the environment and influences the organism’s adaptation to the specific environmental conditions, affecting phenotypic results. Transcriptomics [13] focuses on detecting and quantifying the gene expression of genome coding regions (i.e., mRNA) concerning a specific tissue or cell [14,15]. Proteomics [16,17] focuses on detecting proteins and analyzing their specific interactions, which often confer phenotypic results. Other omics, such as metagenomics [18], focus on sequencing and analyzing environmental samples, allowing us to further discover and understand the microbial communities associated with human niches and their potential correlation to human health and diseases [7,19,20,21,22,23,24,25,26]. Metabolomics [27] dives deeper into proteomics, enabling the study and analysis of metabolites and their influence on the organism’s functional molecular pathways.

Nonetheless, the selection of the most appropriate omics approach can take into consideration the evolutionary timescale [8]. Moreover, in 2024, a novel, promising cell-focused method named “expansion in situ genome sequencing” could bring new insights into how proteins and metabolites interact in their natural environment [28].

In any case, novel bioinformatics tools are necessary to enable and further improve the analysis of such big datasets to fill the necessary gaps (e.g., [29,30,31]). Their application often begins at the sequencing stage and proceeds downstream to the end results. In genomic medicine, much attention has been paid to the final stages of variant data analysis and interpretation, where there is greater difficulty in accurately detecting causal pathogenic variants, which are subject to many known (e.g., [6]) and unknown biochemical forces and factors influencing, for instance, the expression of RNAs, or disrupting protein sites and their conformation. Many of these bioinformatics methods involve criteria and knowledge frequently associated with other areas, such as evolutionary genomics, and more specifically, sequence or site conservation, transitions/transversion ratios (Ka/Ks), sequence homology, etc. (e.g., [3,6,13,32,33,34]). Curiously, in either field, mutations are the cause of fitness [17] and disease [4]. Considering that “nothing makes sense except in the light of evolution” [35], such evolutionary considerations would also make sense when applied to genomic medicine [32]. This would also mean that evolutionary bioinformatics and omics methods could be further devised, improved, and adapted to highlight and improve the detection of the pathogenic molecular changes causing diseases [32], hereon at the population scale [6], or perhaps at the individual personalized scale.

### 3.2. Gap in Knowledge

Given the potential for detecting the underlying causes of diseases, it becomes relevant to provide a dedicated Special Issue chiefly focused on conditions of Mendelian nature. However, this frequently requires the development and improvement of novel omics and/or bioinformatics methods. The first steps in considering evolution in genomic medicine have been taken [3,6,32], but it becomes interesting to understand how far biomedical scientific advances can reach by introducing more from evolutionary biology into medicine [36,37].

Moreover, it would be interesting to understand how these pathogenic variants are causing diseases, what is their source, why are they being created. Could they be regarded as part of natural selection processes? It is well known that among others, they may be due to environment, food habits, microbiome, and/or genetics changes [6,34]. Regarding the microbiome, there are evidences of the influences of the microbial life affecting human genetics and health [6], such as those from genotoxicity [24,25], dysbiosis [20,22,26], and even of viral influence (e.g., [38]). Thus, the application of different omics approaches are paramount to decipher the underlying causes, but also to unravel appropriate and novel therapeutic strategies.

### 3.3. Special Issue Results

Several omics approaches (WES, epigenetics, proteomics, and GWAS) have been applied to the analyses, diagnosis, and potential treatments of diverse diseases, ranging from physical to mental maladies. However, there is a growing need for further contributions employing WGS, transcriptomics, metagenomics, and metabolomics, linking evolutionary, comparative approaches, and the development of bioinformatics software for data analysis. This could become quite useful, for instance, in microbial genomics/metagenomics focused on human interactions or symbiosis, as well as genotoxicity, thus fulfilling our initial “aims and scope” (see Section 1, Recalling the Special Issue Aims and Scope). Nevertheless, this Special Issue has made space for different works, as demonstrated by the five published articles.

The contribution of WES enabled the detection of novel causal variants in a Saudi family and the application of methods from evolutionary genomics, such as multiple sequence alignment and phylogeny, using diverse species to corroborate the molecular locations of the causative variation.

The epigenetics contribution enabled to establish markers associated with PTSD, enabling to guide novel research in mental maladies, whose symptoms could be propagated to the descendants/offspring.

Two proteomics-related contributions studied the molecular interactions of proteins (considering sequence conservation, protein homology modeling, molecular docking, etc.) with other proteins and with small molecules, with potential applications in novel strategies regarding the study of protein interactions, in the interpretation of deleterious variants (e.g., [5]), and novel therapeutics.

The last contribution focused on applying GWAS to sensitive skin condition, which, despite not being recognized as a disease, causes discomfort to those presenting the symptoms. This work uncovered molecular changes associated with genes known to be related to the SS symptoms and diagnosis. Furthermore, it could pave the way in establishing how this condition is regarded or classified by the authorities, and enable further potential personalized treatments.

### 3.4. Future Work

There is room to consider more publications to fulfill the overall objective of this Special Issue, which we aim to respond in the future. Additional research could focus on omics and diseases not here covered. Moreover, potential integrative data analyses [39], or multi-omics, are gradually becoming more cost-effective, and could help to increase the capacity for novel analyses, methods, and bioinformatics applications, and with this, the capacity to also improve the accuracy in the discovery of diseases’ molecular bases [7]. In fact, novel multi-omics [40,41] strategies are becoming everyday more and more in demand, as the isolated omics application have been found to offer limited results, rendering them insufficient in light of the wider spectrum of multiple disease-specific causes and factors to be investigated arising from the progressive study of diseases [6,39].

## 4. Conclusions

This Special Issue provided room for disruptive research considering not only data analyses, but also bioinformatics methods required to facilitate and improve the results of omics analyses. Our results show that important publications are disruptive within their own scopes, thus contributing to biomedical advances and therapeutics. They targeted diverse diseases, such as FH, PTSD, under/overgrowth syndromes, cardiac diseases, neurodegenerative diseases, and SS, and consider certain aspects of evolutionary genomics. In its second volume [40,41], we hope to expand these contributions to the multi-omics scale with a broader evolutionary influence and by publishing manuscripts in a Joint Special Issue between *Life* and *Computation*, wherein the latter journal now provides a dedicated scope for bioinformatics and computational biology contributions.
